# Frequency-Dependent Spatial Distribution of Functional Hubs in the Human Brain and Alterations in Major Depressive Disorder

**DOI:** 10.3389/fnhum.2019.00146

**Published:** 2019-05-14

**Authors:** Anja Ries, Matthew Hollander, Sarah Glim, Chun Meng, Christian Sorg, Afra Wohlschläger

**Affiliations:** ^1^Department of Neuroradiology, Technical University of Munich (TUM), Munich, Germany; ^2^TUM-Neuroimaging Center, Technical University of Munich (TUM), Munich, Germany; ^3^Graduate School of Systemic Neurosciences, LMU Munich, Munich, Germany; ^4^Department of Psychiatry, University of Cambridge, Cambridge, United Kingdom; ^5^Department of Psychiatry, Technical University of Munich (TUM), Munich, Germany

**Keywords:** resting-state fMRI, BOLD, degree centrality, functional connectivity, hubs, major depressive disorder, spectral analysis

## Abstract

Alterations in large-scale brain intrinsic functional connectivity (FC), i.e., coherence between fluctuations of ongoing activity, have been implicated in major depressive disorder (MDD). Yet, little is known about the frequency-dependent alterations of FC in MDD. We calculated frequency specific degree centrality (DC) – a measure of overall FC of a brain region – within 10 distinct frequency sub-bands accessible from the full range of resting-state fMRI BOLD fluctuations (i.e., 0.01–0.25 Hz) in 24 healthy controls and 24 MDD patients. In healthy controls, results reveal a frequency-specific spatial distribution of highly connected brain regions – i.e., hubs – which play a fundamental role in information integration in the brain. MDD patients exhibited significant deviations from the healthy DC patterns, with decreased overall connectedness of widespread regions, in a frequency-specific manner. Decreased DC in MDD patients was observed predominantly in the occipital cortex at low frequencies (0.01–0.1 Hz), in the middle cingulate cortex, sensorimotor cortex, lateral parietal cortex, and the precuneus at middle frequencies (0.1–0.175 Hz), and in the anterior cingulate cortex at high frequencies (0.175–0.25 Hz). Additionally, decreased DC of distinct parts of the insula was observed across low, middle, and high frequency bands. Frequency-specific alterations in the DC of the temporal, insular, and lateral parietal cortices correlated with symptom severity. Importantly, our results indicate that frequency-resolved analysis within the full range of frequencies accessible from the BOLD signal – also including higher frequencies (>0.1 Hz) – reveals unique information about brain organization and its changes, which can otherwise be overlooked.

## Introduction

Major depressive disorder (MDD) has been ranked as one of the most burdensome diseases across the world in terms of total disability-adjusted life years (Murray et al., 2012), affecting various aspects of life and work in more than 300 million people worldwide (World Health Organization, 2017). MDD is characterized by single or recurrent major depressive episodes during which patients experience depressed mood, impaired cognition, energy loss, vegetative symptoms, and suicidal thoughts (American Psychiatric Association [APA], 2000). Currently, the predominant method of diagnosis in MDD is based on psychiatric interviews and patients’ self-reports, and there is still need for more objective and quantifiable procedures. The ever-increasing amount of brain imaging data viewing depression highlights the relevance of the functional organization of large-scale brain systems in MDD pathophysiology, attempting to reveal an informative and putatively more accurate method for mental illness diagnosis and treatment (Drysdale et al., 2016) which could supplement the current diagnostic procedures.

Blood oxygen level dependent (BOLD) time series at rest, measured by functional magnetic resonance imaging (fMRI) at ultra-slow frequencies (<1 Hz), exhibit correlated activity between anatomically remote brain regions – known as functional connectivity (FC; Friston et al., 1996; Horwitz, 2003). Consistent patterns of FC have been observed and categorized into different resting-state networks (RSNs; Biswal et al., 1995; Greicius et al., 2003; Fox et al., 2005; Fox and Raichle, 2007) which constitute a powerful tool to investigate large-scale brain organization. Core RSNs include the default-mode network (DMN; Raichle et al., 2001; Buckner et al., 2008), salience network (SN; Seeley et al., 2007; Menon and Uddin, 2010), and central executive network (CEN; Seeley et al., 2007; Vincent et al., 2008). Alterations in FC patterns both within and between several networks have been implicated in a number of neurological disorders (Badhwar et al., 2017; Dong et al., 2018; Gürsel et al., 2018) – including MDD (Greicius et al., 2007; Sheline et al., 2009; Veer et al., 2010; Menon, 2011; Northoff et al., 2011; Zeng et al., 2012b; Manoliu et al., 2013; Belleau et al., 2015). Although resting-state FC alterations in MDD have been thoroughly reviewed (Wang et al., 2012; Dutta et al., 2014; Dichter et al., 2015; Mulders et al., 2015) and several meta-analyses have been performed (Kaiser et al., 2015; Zhong et al., 2016), most studies that reported FC alterations operate on specific assumptions and methodological constraints, which in this study we aim to extend in a twofold manner.

Firstly, previous studies mostly derive FC patterns from data that were band-pass filtered beforehand, mainly focusing on the conventional low-frequency range of 0.01–0.1 Hz (Biswal et al., 1995; Lowe et al., 1998; Cordes et al., 2001; Cordes et al., 2002; Greicius et al., 2003; Fox and Raichle, 2007). Such approach limits observations on BOLD fluctuations to a narrow frequency range and putatively overlooks important information about higher frequency bands. Recent studies on spectral properties of RSN BOLD fluctuations show that spatial patterns of characteristic networks can be detected at multiple frequency bands, including high frequencies up to 0.25 Hz or even 0.75 Hz (Niazy et al., 2011; Van Oort et al., 2012; Boubela et al., 2013; Gohel and Biswal, 2015). Thus, meaningful information of neural origin can be obtained from a broader frequency range of BOLD fluctuations than previously assumed, given the signal from non-neural sources is correctly accounted for.

Secondly, FC patterns are mostly analyzed across a single broad frequency range, without distinction into specific sub-bands. However, several studies highlight the importance of frequency-specific BOLD signal analysis. Distinct brain regions were shown to operate on distinct timescales of intrinsic (i.e., resting-state) activity (Baria et al., 2011; Honey et al., 2012; Stephens et al., 2013; Murray et al., 2014). FC within and between networks, as well as the resulting network topologies, were shown to be governed in a frequency specific manner (Salvador et al., 2008; Wu et al., 2008; Zuo et al., 2010; Sasai et al., 2014; Thompson and Fransson, 2015; Gollo et al., 2017). Furthermore, a relation between the frequency regime and the directionality of information flow has been made (Neufang et al., 2014; Cocchi et al., 2016). Moreover, De Domenico et al. (2016) proposed that brain activity and topology should be analyzed in a multiplex fashion, where all information obtained from different frequency bands is considered simultaneously. Hence, the human brain is a complex system, that operates on multiple timescales. Neural signals detected at different frequency bands may originate from different oscillators, with specific properties and physiological functions (Penttonen and Buzsáki, 2003; Draguhn and Buzsáki, 2004; Knyazev, 2007). Altogether, empirical evidence highlights the importance of frequency information when investigating functional brain organization.

While previous studies on FC changes within one frequency regime in MDD have proven to be informative, here we argue that it is essential to implement frequency-specific analyses to better understand the underlying changes in connectivity in MDD and their implications for brain function. More recently, studies on MDD have addressed the needs for frequency-dependent analysis of brain organization at rest and observed frequency specific alterations in local and global brain topology (Luo et al., 2015), regional homogeneity (Xue et al., 2016), and amplitude of low-frequency oscillations (Wang et al., 2016). However, when subdividing the BOLD bandwidth into distinct sub-bands, the study of Xue et al. (2016) operates on the conventional, narrow frequency band of 0.01–0.08 Hz and as such does not consider higher frequencies. The studies of Luo et al. and Wang et al. – although they do consider higher frequencies (i.e., >0.1 Hz) – use rather broad frequency intervals when dividing the BOLD signal into distinct frequency sub-bands. These frequency sub-bands were adapted from the nomenclature of Buzsáki and colleagues, who defined the slow-5 (0.01–0.027 Hz), slow-4 (0.027–0.073 Hz), slow-3 (0.073–0.198 Hz) and slow-2 (0.198–0.25 Hz) frequency bands based on electrophysiological recordings but not from the BOLD signal itself (Penttonen and Buzsáki, 2003; Draguhn and Buzsáki, 2004). Thus, these frequency bands might not constitute the optimal division scenario for the rs-fMRI signal (Thompson and Fransson, 2015). In the current study, we divide the BOLD bandwidth into distinct frequency sub-bands at a much more fine-grained spectral resolution (i.e., using frequency intervals of 0.025 Hz), and determine frequency-specific FC connectivity patterns at a greater resolution.

Degree centrality (DC) reflects the overall connectedness of one brain voxel to the rest of the brain (Buckner et al., 2009; Tomasi and Volkow, 2010; Takeuchi et al., 2015). Hubs – i.e., highly connected brain regions – play an important role in information integration and flow by mediating interactions among distinct brain regions (Fransson and Marrelec, 2008; Buckner et al., 2009; Zuo et al., 2012; Nijhuis et al., 2013; Power et al., 2013). High-centrality hubs entail higher energetic and metabolic demands (Bullmore and Sporns, 2012), possess the highest level of neuronal activity (de Haan et al., 2012), and constitute points of increased vulnerability to brain damage and neurodegenerative disorders (Buckner et al., 2009; Sperling et al., 2009; Crossley et al., 2014). Thus, hub distribution and their dysfunction could contribute to neuropsychiatric diseases.

In this study, we investigate FC patterns of BOLD signal fluctuations at 10 distinct frequency sub-bands from the full range of accessible frequencies (i.e., 10 sub-bands obtained from the full frequency range of 0.01–0.25 Hz, each of them with a bandwidth of 0.025 Hz) in healthy subjects and look for FC alterations at each frequency band in MDD patients. We use the DC as a proxy of FC. We hypothesize that the spatial distribution of hubs varies between different frequency bands in healthy controls, and that MDD has characteristic impacts on frequency-specific hubness.

Additionally, we examine DC patterns within the broad range of accessible frequencies taken as a single band (i.e., 0.01–0.25 Hz), as well as within the “conventional” frequency band (i.e., 0.01–0.1 Hz) previously considered by the majority of rs-fMRI studies.

**Table 1:** Demographic and clinical characteristics.

	MDD (*n* = 24)	HC (*n* = 24)	MDD vs. HC^a,b^

	Mean (*SD*)	Mean (*SD*)	*P*-value
Age (years)	48.25 (14.92)	43.63 (14.91)	>0.05^a^
Gender (m/f)	11/13	10/14	>0.05^b^
Duration of MDD (years)	16.92 (10.38)	NA	
Number of episodes	5.46 (2.47)	NA	
Duration of current episode (weeks)	16.38 (6.70)	NA	
GAF	50.17 (10.60)	99.50 (1.10)	<0.001^a,∗^
HAM-D	21.83 (7.06)	0	<0.001^a,∗^
BDI	23.58 (5.92)	0	<0.001^a, ∗^

*^a^two-sample t-test. ^b^χ^2^-test. ^∗^significant for p < 0.05, Bonferroni-corrected for multiple comparisons. Abbreviations: MDD, major depressive disorder; HC, healthy controls; SD, standard deviation; GAF, Global Assessment of Functioning Scale; HAM-D, Hamilton Depression Rating Scale; BDI, Beck Depression Inventory.*

## Materials and Methods

### Subjects

This study included 25 healthy control (HC) subjects and 25 patients who suffered from recurrent MDD. Participants’ data have also been used in previous studies (Manoliu et al., 2013; Meng et al., 2014; Ries et al., 2018). One HC subject and one MDD patient had to be excluded due to spatial normalization problems which occurred during correction for physiologic noise and motion, resulting in a total of 24 HC subjects and 24 MDD patients taken into further analysis (see Table 1 for detailed information on demographical and clinical characteristics).

Patients with MDD were recruited from the Department of Psychiatry of the Klinikum rechts der Isar, Technische Universität München, by psychiatrists. HC subjects were recruited via advertising. All participants provided informed consent in accordance with the Human Research Committee guidelines of the Klinikum rechts der Isar, Technische Universität München. All participants were examined for their medical history, underwent psychiatric interviews and psychometric assessments. Psychiatric diagnoses were based on Diagnostic and Statistical Manual of Mental Disorders-IV (DSM-IV; American Psychiatric Association [APA], 2000). The Structured Clinical Interview for DSM-IV (SCID) was used to assess the presence of psychiatric diagnoses (First et al., 1996). The severity of depression symptoms was measured with the Hamilton Rating Scale for Depression (HAM-D; Hamilton, 1960), as well as the Beck Depression Inventory (BDI; Beck et al., 1961). The global level of social, occupational, and psychological functioning was measured with the Global Assessment of Functioning Scale (GAF; Spitzer et al., 1992). The clinical-psychometric assessment was performed by psychiatrists who have been professionally trained for SCID interviews with inter-rater reliability for diagnoses and scores of >95%. Recurrent MDD was the primary diagnosis for all patients. All patients met the criteria for a current major depressive (MD) episode, where the average episode length was 16.38 weeks (*SD* = 6.70), the average HAM-D score was 21.83 (*SD* = 7.06), and the average BDI score was 23.58 (*SD* = 5.92). The mean duration of MDD was 16.92 years (SD = 10.38), with a mean number of episodes of 5.46 (SD = 2.47). The average GAF-score was 50.17 (SD = 10.60). The mean duration of MDD was 16.92 years (*SD* = 10.38), with a mean number of episodes of 5.46 (*SD* = 2.47). The average GAF-score was 50.17 (*SD* = 10.60). Fourteen MDD patients had psychiatric co-morbidities, including generalized anxiety disorder (*n* = 6), avoidant or dependent personality disorder (*n* = 5), and somatization disorder (*n* = 3). Exclusion criteria for the patients included psychotic symptoms, schizophrenia, schizoaffective disorder, bipolar disorder, and substance abuse. Additional exclusion criteria for both groups were pregnancy, neurological or severe internal systemic diseases, and general contraindications for MRI. One MDD patient was not undergoing psychotropic medication treatment by the time of MRI assessment. Seven patients were treated by antidepressant mono-therapy [three cases: citalopram 30 mg/d (mean dose); three cases: sertraline 200 mg/d; one case: mirtazapine 30 mg/d); 11 patients by dual-therapy (five cases: citalopram 37.5 mg/d + mirtazapine 30 mg/d; two cases: citalopram 40 mg/d + venlafaxine 225 mg/d; one case: citalopram 30 mg/d + quetiapine 200 mg/d; one case: sertraline 200 mg/d + mirtazapine 30 mg/d; two cases: venlafaxine 225 mg/d + mirtazapine 30 mg/d); and five patients by triple-therapy (two cases: citalopram 30 mg/d + venlafaxine 187.5 mg/d + amisulpride 200 mg/d; two cases: citalopram 30 mg/d + mirtazapine 30 mg/d + quetiapine 200 mg/d; 1 case: venlafaxine 22 mg/d + mirtazapine 30 mg/d + quetiapine 200 mg/d]. All HC subjects were free of any current or past neurological or psychiatric disorder or psychotropic medication.

### Data Acquisition and Preprocessing

All participants underwent 10 min of a resting-state functional MRI (rs-fMRI) scan, for which they were instructed to keep their eyes closed, not to fall asleep, and not to think of anything. Subjective verification that participants stayed in a state of alertness during the rs-fMRI scan was obtained by interrogating them via intercom immediately after the scan. All participants successfully completed the scanning sessions.

#### Data Acquisition

MRI data were collected on a 3-Tesla Philips Achieva scanner with an 8-channel phased-array head coil. T1-weighted structural images were acquired with an MPRAGE sequence (echo time = 4 ms, repetition time = 9 ms, inversion time = 100 ms, flip angle = 5°, field of view = 240 mm × 240 mm, matrix = 240 × 240, 170 slices, slice thickness = 1 mm, and 0 mm interslice gap, voxel size = 1 mm × 1 mm × 1 mm). Functional MRI data were obtained by using a gradient echo EPI sequence (echo time = 35 ms, repetition time = 2000 ms, flip angle = 82°, field of view = 220 mm × 220 mm, matrix = 80 × 80, 32 slices, slice thickness = 4 mm, and 0 mm interslice gap, voxel size = 2.75 mm × 2.75 mm × 4 mm; 300 volumes).

#### Physiologic Noise and Motion Correction

Physiologic Estimation by Temporal ICA (PESTICA^1^; Beall and Lowe, 2007) was applied on raw fMRI data to detect the pulse and breathing cycles in individual subjects. Peak frequencies of cardiac and respiratory rate time courses obtained from PESTICA (with a temporal resolution of TR/slice number = 2s/32) were calculated using Fast Fourier Transform (FFT) in Matlab. Precisely, a Gaussian fit in a search window was applied which corresponded to expectation values for the physiological rhythms [cardiac 55–70bpm (beats per minute); respiratory 10–24 bpm]. For quality assurance, visual check of fit quality was performed. Differences in cardiac and respiratory rates between groups were tested with two-sample *t*-tests. The tests yielded no significant difference between the groups regarding the cardiac rate (*p* > 0.5), as well as the respiratory rate (*p* > 0.5).

Subsequently, the PESTICA-monitored pulse and respiration cycles were used for retrospective image correction (RETROICOR). RETROICOR is an image-based correction method, in which low-order Fourier series are fit to image data based on the time of each image acquisition relative to the phase of the cardiac and respiratory cycles (Glover et al., 2000). The method results in the voxel level removal of physiologic noise from the data.

Following the physiologic noise correction, motion corruption was accounted for using slice-oriented motion correction (SLOMOCO; Beall and Lowe, 2014). This method uses an algorithm to estimate the out-of-plane motion and correct the data using the acquired estimates. SLOMOCO has been shown to perform better than volumetric methods and to precisely detect motion of independent slices, correcting for most all effects of motion corruption in BOLD data. Differences in SLOMOCO estimates of head movement between groups were tested, to further control for head motion effects across groups. The mean relative head displacement was calculated as the root mean squared volume-to-volume displacement (Satterthwaite et al., 2013; Power et al., 2014). A two-sample *t*-test revealed no significant differences in mean relative head motion between groups (*p* > 0.05).

#### Normalization

The data which were controlled for physiological and motion effects were subsequently normalized into the Montreal Neurological Institute (MNI) standard space using statistical parametric mapping software SPM12^2^.

#### Nuisance Covariates Regression

To further reduce the impact of non-neural sources on the BOLD signal, the regression of nuisance covariates including white matter (WM) and cerebrospinal fluid (CSF) was performed. For each participant, binarized region of interest (ROI) masks of the WM and CSF were created from T1 segmentation compartments (binarized at a threshold of 0.9). Averaged CSF and WM signals were extracted separately from individual participants data and served as covariable signals. Nuisance covariate regression was performed on the previously realigned and normalized, but not smoothed data, using Resting-State fMRI Data Analysis Toolkit (REST V1.8^3^). Subsequently, data were spatially smoothed in SPM12 using a 6-mm full width at half maximum (FWHM) Gaussian kernel.

## Data Analysis

### Degree Centrality Maps

Degree centrality maps were computed for each participant by use of the REST toolbox which implements an approach similar to that proposed by Buckner et al. (2009) and Zuo et al. (2012). For this analysis, the smoothed data with nuisance covariates regression were used. The procedure for the DC calculation is the following: for each voxel, a correlation coefficient between its own time course and the time course of every other voxel in the brain is calculated. This results in a connectivity map of the given voxel which, in the next step, is binarized at a selected threshold (here, *r* = 0.2). During binarization, all correlation coefficients below the given threshold are set to zero, while all correlation coefficients above the threshold are set to 1. In the last step, the sum of all non-zero connections for the given voxel is calculated. This procedure is repeated for all voxels, yielding a whole-brain DC map. The single-subject DC maps are then normalized by applying a z-transform, i.e., subtracting the mean and dividing by the standard deviation of the degree across all voxels within the brain.

Degree centrality maps were calculated (i) within 10 frequency bands (freq1: 0.01–0.025; freq2: 0.025–0.05; freq3: 0.05–0.075; freq4: 0.075–0.1; freq5: 0.1–0.125; freq6: 0.125–0.15; freq7: 0.15–0.175; freq8: 0.175–0.2; freq9: 0.2–0.225; freq10: 0.225–0.25 Hz), (ii) within the full frequency range (0.01–0.25 Hz), as well as (iii) within the conventional frequency range (0.01–0.1 Hz). The BOLD signal was band-pass filtered into individual frequency bands using REST toolbox, which by default uses the ideal rectangle window. In REST, the ideal filter transforms the time series into the frequency domain via the discrete Fourier transform and adds zeros to extend the frequency coverage, then transforms back to the time domain by using the inverse discrete Fourier transform (Song et al., 2011).

### Construction of Gray Matter Volume Covariates: Brain Volumetric Analysis

Reduction in gray matter volume (GMV) can affect measures of FC such as the DC. Particularly, decreased GMV in a ROI results in higher contribution of CSF on the signal variance recorded from the corresponding brain region and leads to increased noise in the signal. Measures of FC, such as the correlation coefficient, are highly susceptible to noise (Dagli et al., 1999; Birn et al., 2006; Shmueli et al., 2007; Chang et al., 2009; Power et al., 2012; Van Dijk et al., 2012). The study of Dukart and Bertolino (2014) showed that up to 25% of within-subject variance in DC can be explained by the underlying structural information, where lower DC has been observed in brain regions with higher contributions of non-GM tissues. Thus, it is important to control for the effect of between-group differences in GMV on FC metrics.

Voxel-based morphometry (VBM) analysis was performed using the Computational Anatomy Toolbox (CAT12^4^) implemented in SPM12 to detect brain volumetric differences between groups. The procedure was the following: structural T1 images were normalized to a template space and later segmented into GM, WM, and CSF. To assure image quality, resulting images were inspected for homogeneity. None of the images had to be removed. As part of the CAT12 workflow, for each participant the (i) total intracranial volume (TIV) and (ii) absolute as well as relative global GM, WM, and CSF volumes were estimated. Next, the resulting GM images were smoothed via SPM12 module using a Gaussian kernel with a FWHM of 8 mm. Group differences between smoothed GM volumes were tested using voxel-wise two-sample *t*-tests in SPM12 (*p* cluster-level corrected <0.05, on underlying voxel-level correction of *p* < 0.001), controlling for individual TIV values.

The complete set of the subjects’ GMV images could be converted to voxel-wise regressors on the group level ready to be included in the subsequent analysis of DC.

### Statistical Analysis

Using SPM12, single-subject DC maps of the HC group were subjected to one-sample *t*-tests (*p* cluster-level corrected < 0.05, on underlying voxel-level correction of *p* < 0.001, with voxels restricted to GM) to define the healthy DC distribution at each of the 10 frequency bands, as well as at the full frequency range (i.e., without division into sub-bands) and at the conventional frequency range. Next, in HC the similarity between the group averaged DC map obtained from the BOLD signal at the full frequency range and the DC maps at 10 frequency sub-bands was examined. To this end, cross-correlations were calculated between the group averaged DC map at the full frequency range and the group averaged DC maps at each of the 10 frequency bands down-sampled to 0.4 of the resolution in order to match the intrinsic smoothness of the data (Pearson’s correlation, *N* = 5091). Frequency sub-band of maximum correlation was determined and statistically compared to all other frequency sub-bands based on Pearson’s *r* values and the degrees of freedom.

Potential differences in DC associated with groups or frequency sub-bands were examined within a flexible factorial design, with the main factors group and frequency sub-band, followed by *t*-tests (*p* cluster-level corrected < 0.05, on underlying voxel-level correction of *p* < 0.001, with voxels restricted to GM).

Additionally, two-sample *t*-tests were performed on DC maps obtained from (i) the full frequency range without distinction into specific sub-bands and (ii) the conventional frequency band to test for group differences (*p* cluster-level corrected < 0.05, on underlying voxel-level correction of *p* < 0.001, with voxels restricted to GM).

To control for group differences in DC possibly resulting from the GM atrophy in MDD patients, we performed a whole-brain voxel-wise regression between the individual DC maps and the resliced GM volumes at each frequency sub-band using a Matlab based in-house script. Regression residuals constituted the new, GMV-corrected DC maps. Subsequently, group differences in the GMV-corrected DC maps were examined at each frequency sub-band within a flexible factorial design with the main factors group and frequency sub-band, and were tested with *t*-tests (*p* clusterlevel corrected < 0.05, on underlying voxel-level correction of *p* < 0.001, with voxels restricted to GM). Results of this analysis are presented in the Supplementary Material.

### Correlation Between DC and Symptom Severity

Clusters reflecting significant group differences in DC at individual frequencies served as ROIs for the extraction of DC values at the subject level. For each frequency sub-band where a significant group difference was found, the averaged DC value for each participant was extracted in a cluster-wise manner. Pearson’s correlation was calculated between the averaged DC values for each cluster at each frequency sub-band and the symptom severity scores (with Bonferroni-correction for multiple comparisons, *n* = 9 only frequencies at which significant group differences in DC were found). In a *post hoc* analysis, we investigated whether the observed correlations occur independently of the reductions in GMV. From each cluster where a significant correlation between the averaged DC and the symptom characteristics was found, averaged GMV-controlled DC values were extracted in addition and were correlated with symptom characteristics.

## Results

### Brain Volumetric Analysis

Compared to HC subjects, MDD patients exhibited regional GM atrophy (displayed in Supplementary Figure S1). Specifically, decreased GMV was found bilaterally in the middle frontal gyrus (Supplementary Figure S1: clusters 1 and 2); the right middle orbital gyrus (Supplementary Figure S1: cluster 1); the left anterior cingulate cortex (ACC) and left superior medial frontal gyrus (Supplementary Figure S1: cluster 3); as well as in the left hippocampus and left superior temporal gyrus (Supplementary Figure S1: cluster 4). Detailed information about cluster peaks, their coordinates, *z*-values and anatomical locations are presented in Supplementary Table S1.

### Frequency-Specific Degree Centrality

**FIGURE 1:**
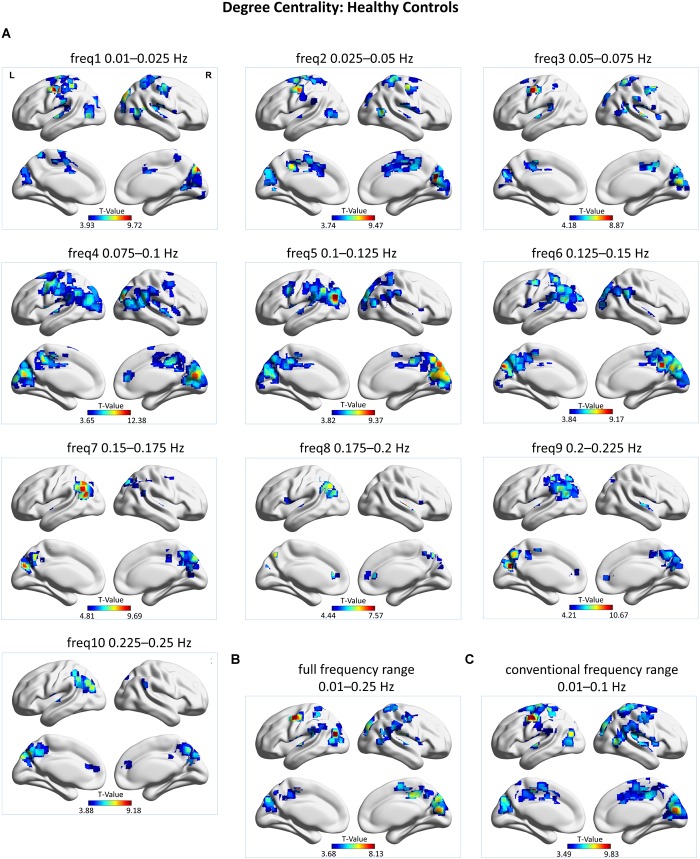
Group-level voxel-wise degree centrality (DC) maps for healthy control (HC) subjects. DC maps were obtained by subjecting individual z-maps to a one-sample *t*-test (*p* cluster-level corrected < 0.05, on underlying voxel-level correction of *p* < 0.001) with voxels restricted to gray matter. **(A)** DC maps at 10 different frequency bands, represented by *t*-values. **(B)** DC map at the full frequency range of 0.01–0.25 Hz, represented by *t*-values. **(C)** DC map at the conventional frequency range of 0.01–0.1 Hz, represented by *t*-values. L = left, R = right. DC clusters were visualized with the BrainNet Viewer (http://www.nitrc.org/projects/bnv/; Xia et al., 2013).

Group-level DC maps were obtained from HC subjects in a frequency-resolved fashion (i.e., at 10 separate frequency sub-bands), as well as at the full frequency range (i.e., 0.01-0.25 Hz) and the conventional frequency range (i.e., 0.01–0.1 Hz), and are displayed in Figure 1. A frequency-specific spatial distribution of hubs – i.e., regions displaying high DC – is evident. A detailed list of frequency-dependent hubs, as well as hubs occuring within the full frequency regime and the conventional frequency regime is presented respectively in Supplementary Tables S2a–c.

Overall, in HC subjects, hub regions were found to be distributed in a frequency-specific manner (see Figure 1A and Supplementary Table S2a). At low-frequency bands (freq1–3; 0.01–0.075 Hz) high DC voxels were reported in the cuneus, post- and precentral gyri, paracentral lobule, superior temporal gyrus, and middle cingulate cortex (MCC). Additionally, at freq1 the insula was also found to exhibit high DC. At middle-frequency bands (freq4–7; 0.075–0.175 Hz) the middle occipital gyrus, cuneus, precuneus, MCC, angular gyrus, inferior parietal lobule (IPL), supramarginal gyrus, and the insula exhibited high DC. At high-frequency bands (freq8–10; 0.175–0.25 Hz), high DC voxels were systematically observed in the cuneus, precuneus, angular gyrus, MCC, IPL, and ACC. Athwart a wide range of frequency bands, the cuneus and MCC as well as the precuneus, IPL, angular gyrus, and supramarginal gyrus appear as crucial regions for information integration – as defined by their high DC profile. Specifically, regions within the lateral parietal cortex appear central at a wide range of frequency bands (i.e., freq3–10; 0.075–0.25 Hz), but most prominently at the middle- to high-frequency bands (i.e., 6–10; 0.125–0.25 Hz).

Degree centrality analysis of the rs-fMRI signal at the full frequency range (i.e., 0.01–0.25 Hz), without decomposition into frequency sub-bands, revealed the following hub regions: the cuneus, precuneus, MCC, paracentral lobule, post- and precentral gyri, middle temporal gyrus, superior temporal gyrus, middle occipital gyrus, angular gyrus, IPL, and insula (see Figure 1B and Supplementary Table S2b).

Degree centrality analysis of the rs-fMRI signal at the conventional frequency range (i.e., 0.01–0.1 Hz) revealed the following hub regions: the cuneus, precuneus, MCC, paracentral lobule, post- and precentral gyri, middle temporal gyrus, superior temporal gyrus, middle occipital gyrus, superior parietal lobule, IPL, supramarginal gyrus, superior frontal gyrus, middle frontal gyrus, and insula (see Figure 1C and Supplementary Table S2c).

Correlation analysis was carried out between the group averaged DC map at the full frequency range and the DC maps at each of the 10 frequency sub-bands. Results revealed the highest correlation between the DC map at full frequency range and the DC map at frequency band 3 (freq1: *r* = 0.6392, freq2: *r* = 0.6472, freq3: *r* = 0.7362, freq4: *r* = 0.6554, freq5: *r* = 0.6127, freq6: *r* = 0.5731, freq7: *r* = 0.3951, freq8: *r* = 0.2664, freq9: *r* = 0.3053, freq10: *r* = 0.3109; *p* < 0.001 Bonferroni-corrected for multiple comparisons). The correlation was significantly higher than the one of any other frequency sub-band toward the DC map at full frequency range (*p* < 0.001, corrected for multiple comparisons, Bonferroni factor = 45).

**FIGURE 2:**
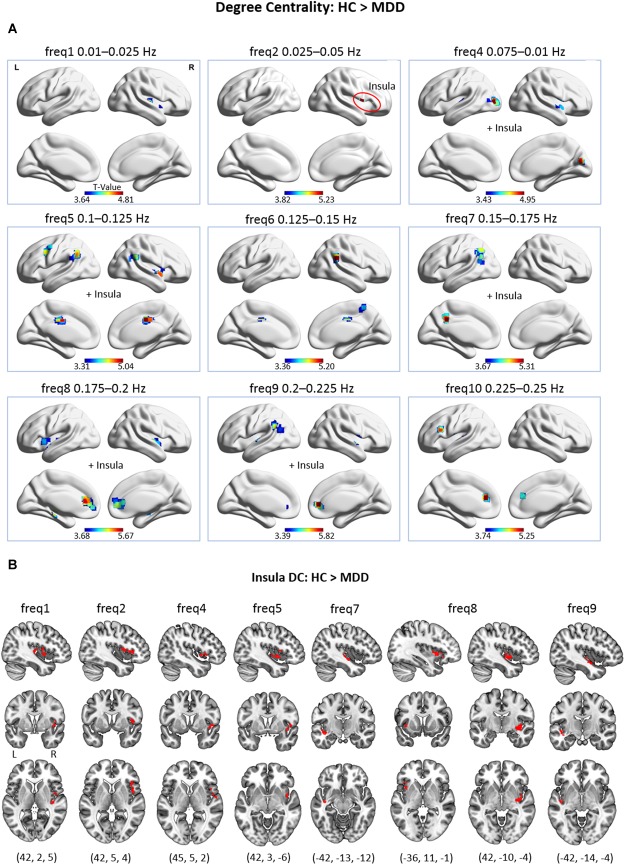
**(A)** Group differences in voxel-wise DC across 9 frequency bands, as only the frequency bands where significant differences were found are displayed (*p* cluster-level corrected < 0.05, on underlying voxel-level correction of *p* < 0.001, with voxels restricted to GM). At freq2, the cluster is located exclusively in the insular lobe. **(B)** Group differences in voxel-wise DC of the insular lobe at different frequency bands. This figure encloses Supplementary Information to panel **(A)** for improved visibility.

In MDD, significant frequency-specific differences in regional DC were observed in a wide range of frequency bands (see Figure 2A). A detailed list of brain regions with significantly decreased DC in MDD patients is presented in Supplementary Table S3 of the Supplementary Material. Importantly, one should note that in MDD patients, when compared to HC subjects, no significant increases in DC were found.

Across frequencies, a pattern of brain regions with decreased DC in MDD patients emerges. Particularly, in the low-frequency bands (freq1, 2; 0.01–0.05 Hz) decreased DC of the insula (at freq1, 2) and the transverse temporal gyrus (at freq1) was found in depression. No significant difference was found in low-frequency band 3 (freq3; 0.05–0.075 Hz). In the middle-frequency bands (freq4–7; 0.075–0.175 Hz), the middle occipital gyrus, calcarine gyrus, superior temporal gyrus (at freq4); the insula (at freq4, 5); the MCC and supramarginal gyrus (at freq5, 6); the precuneus (at freq6, 7); and the angular gyrus (at freq5, 7) showed decreased DC in MDD. In the high-frequency range (freq8–10; 0.175–0.25 Hz), decreased hubness of the ACC and the superior temporal gyrus (at freq8–10); the putamen and hippocampus (at freq8); the insula (freq8,9); the supramarginal gyrus and the IPL (at freq9) was observed in MDD.

Notably, the insula was found to exhibit decreased DC in MDD patients across several frequencies (i.e., freq1, 2, 4, 5, 7, 8, and 9). Upon closer inspection, it is visible that peak coordinates representing voxels of decreased DC within the insula to a large extent differ in their exact position across frequencies (see Figure 2B). Thus, in the MDD patient group, largely distinct parts of the insula exhibited reduced DC depending on the frequency band.

The two-sample *t*-tests carried out on the DC maps obtained from the BOLD signal within the full frequency range (i.e., 0.01–0.25 Hz) as well as within the conventional frequency range (i.e., 0.01–0.1 Hz) did not reveal any significant group differences at the given significance threshold.

Subsequently, group differences in DC at distinct frequency bands were examined, controlling for the possible influence of regional GM atrophy in MDD patients. Per subject, estimates of GMV were regressed out from the corresponding DC maps at each frequency band in a voxel-wise manner. Comparable results were obtained when contrasting group GMV-corrected DC maps across frequencies (see Supplementary Figure S2 and Supplementary Table S4).

### Correlation Between DC and Symptom Severity

**FIGURE 3:**
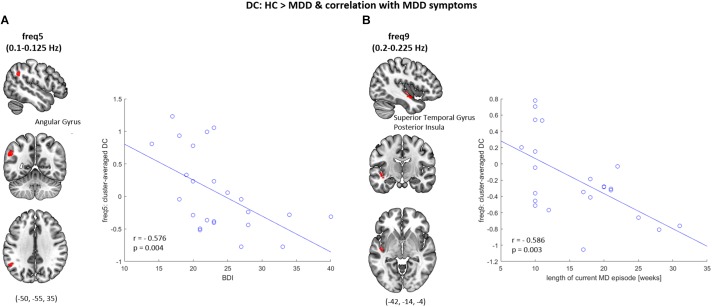
Correlation between the DC and symptom severity. **(A)** At frequency band 5 (freq5: 0.1–0.125 Hz), decreased DC of the left angular gyrus was observed in MDD patients when compared to healthy controls (HC), which significantly correlated with the BDI scores (*r* = −0.576, *p* = 0.004). **(B)** At frequency band 9 (freq9: 0.2–0.225 Hz), decreased DC of the left superior temporal gyrus & posterior insula was observed in MDD patients when compared to HC, which significantly correlated with the length of current major depressive (MD) episode (*r* = −0.586, *p* = 0.003).

Significant correlations between the reduction in regional DC and clinical characteristics of depression were observed (see Figure 3). Specifically, at frequency band 5 (0.1–0.125 Hz), a significant negative correlation between the BDI score and the averaged DC value within a cluster comprising the left angular gyrus was found (*r* = −0.576, *p* < 0.005). At frequency band 9 (0.2–0.225 Hz) a significant negative correlation between the length of the current MD episode and the averaged DC value within a cluster comprising the left superior temporal gyrus and the posterior insula was found (*r* = −0.586, *p* < 0.005). These correlation results are independent of GMV reductions, as they could be replicated in a subsequent correlation analysis which was carried out on the averaged, GMV-corrected DC values from those two clusters (freq5: DC × BDI, *r* = −0.597, *p* < 0.005; freq9: DC × length of current MD episode, *r* = −0.539, *p* < 0.01).

## Discussion

We investigated frequency-resolved regional FC patterns – i.e., degree centrality – determined from the full range of accessible frequencies (0.01–0.25 Hz) of resting-state fMRI signal in healthy controls and MDD patients. Main results indicate a frequency-specific spatial configuration of functional hubs in healthy controls, where the occurrence of dominant hubs gradually shifts from central to posterior regions and is lastly localized in the ACC and parietal-occipital regions, with increasing frequency content. Regions within the lateral parietal cortex retain the role of hubs over a wide range of frequencies. Importantly, the finding of ACC constituting a functional hub at high frequencies (0.175–0.25 Hz) is striking and highlights the importance of consideration of higher frequencies in connectivity analyses, which classically tend to be restricted to frequencies below 0.1 Hz. In major depression, independently of regional GM atrophy, we find frequency-specific alterations in DC of distinct brain regions including the occipital cortex, MCC, sensorimotor cortex, lateral parietal cortex, precuneus, ACC, and the insula.

### Frequency-Specific Spatial Distribution of Hubs in Healthy Control Subjects

The DC is a measure of how well a given brain region is connected to the rest of the brain. Studies of structural (Sporns et al., 2007; Hagmann et al., 2008) and functional (Achard et al., 2006; Power et al., 2013) brain organization revealed a small number of cortical nodes which exhibit an outstandingly high number of connections to other regions. These are referred to as hubs, which play an important role in global communication both by creating short and efficient paths of information flow, and by – dynamically (de Pasquale et al., 2012, 2016, 2017; Kabbara et al., 2017) – supporting information integration across remote brain systems (Colizza et al., 2006; Bullmore and Sporns, 2012). Across studies, specific brain regions have consistently been reported as functional hubs (van den Heuvel et al., 2008; Buckner et al., 2009; Cole et al., 2010; Guye et al., 2010; Tomasi and Volkow, 2010, 2011a,b; Zuo et al., 2012; for review see Kabbara et al., 2017; de Pasquale et al., 2017) and can be attributed to specific RSNs. These are: the medial prefrontal cortex (mPFC), IPL, angular gyrus, and ventral precuneus/posterior cingulate – associated with the DMN; the supplementary motor area, central sulcus and postcentral gyrus – associated with the sensorimotor network; the primary visual cortex and the cuneus – associated with the visual network. From the anatomical perspective, brain regions with dense anatomical connections form structural hubs – which facilitate FC. They include the precuneus/posterior cingulate, superior frontal cortex, medial orbitofrontal cortex, insula, dorsal ACC, and medial temporal cortex, which overlap with all RSNs yet are mostly attributed to the SN and DMN (van den Heuvel and Sporns, 2013).

In the current study, we show that the spatial configuration of functional hubs is frequency-dependent, thus, information integration is governed in a frequency-specific manner over multiple timescales of neural activity. This is in line with previous work revealing frequency-specific functional organization of the brain at rest (Salvador et al., 2008; Sasai et al., 2014; Thompson and Fransson, 2015; Gollo et al., 2017) as well as reports of dynamic processing regime of hubs (de Pasquale et al., 2017; Kabbara et al., 2017). Combined, these studies highlight the flexible and time-varying behavior of hubs, which facilitates information integration across multiple systems at multiple timescales.

In HC subjects, we observed a graded shift in the occurrence of most prominent functional hubs along with increasing frequency content. Precisely, the cuneus, post- and precentral gyri, and paracentral lobule form functional hubs predominantly at lower frequencies (freq1–3; 0.01–0.075 Hz). Middle frequencies (freq4–6; 0.075–0.15 Hz) represent a somewhat intermediate state, where hubs occur widely spread across the brain, but mostly within the middle occipital gyrus and cuneus, as well as the precuneus, angular gyrus, supramarginal gyrus, and IPL. The ACC was found to form a functional hub exclusively at higher frequencies (freq8–10; 0.175–0.25 Hz). Furthermore, the precuneus, angular gyrus, supramarginal gyrus, and IPL were found to exhibit complex connections at higher frequencies (freq7–10; 0.15–0.25 Hz). The insula was found to constitute a hub across several low- and middle-frequency bands.

Hubs defined by the frequency-resolved DC analysis, as well as by the full-band (i.e., 0.01–0.25 Hz) and the conventional band (i.e., 0.01–0.1 Hz) DC analyses, largely correspond to hubs reported in previous studies. The spatial distribution of functional hubs observed at the full frequency range largely resembles the distribution observed at the conventional frequency range. Whereas most of the hubs which were revealed by the frequency-resolved DC analysis could also be detected by the full-band as well as the conventional band DC analyses, there was still unique information about the hub profile of ACC stored in higher frequency bands only, which was not captured by the full-band or the conventional band analyses. The ACC has been identified as a hub before (for review see Table 1 of Kabbara et al., 2017), however, to our knowledge no reports of ACC hubness specific to higher BOLD frequencies exist. This finding stresses the importance of frequency-resolved signal analysis, and the consideration of higher frequencies of resting-state BOLD fluctuations (i.e., >0.1 Hz), as they hold unique information about functional interactions in the brain.

In our study, regions of the lateral parietal cortex such as the IPL, supramarginal gyrus, angular gyrus appear as functional hubs across a wide span of frequency bands (i.e., 0.05–0.25 Hz). These regions form the DMN, which has previously been identified as a key system for information integration across remote networks, since most of the hubs determined from the whole brain can be attributed to the DMN (for review see de Pasquale et al., 2017). Regions of the DMN are, in proportion to hubs of other networks, most widely integrated in the rich club – a strongly interlinked ensemble of highly central brain regions (van den Heuvel and Sporns, 2011). Moreover, the DMN co-forms the dynamic core network (de Pasquale et al., 2013; de Pasquale et al., 2016), whose hubs exhibit a pulsatile pattern of high-centrality states (de Pasquale et al., 2017). Such pulsatile pattern facilitates a highly efficient topological regime for information integration across multiple brain networks in a flexible, time-varying manner. Our finding of a consistent hub profile of regions associated with the DMN across a wide span of frequency bands further corroborates with the notion of the DMN being central for information integration across many functional systems at multiple timescales.

To summarize, previous studies have shown the coexistence of multiple, spatially constrained frequency components within resting-state fMRI fluctuations (Zuo et al., 2010; Baria et al., 2011). Different frequencies of the BOLD signal could reflect distinct physiological processes occurring at different timescales (Draguhn and Buzsáki, 2004; Knyazev, 2007). Our observation of frequency-dependent connectivity patterns could further support the notion of differential oscillatory processes being reflected in the intrinsic BOLD signal (Mantini et al., 2007) and point toward the distinct functional relevance of characteristic hub connectivity profiles at distinct timescales of BOLD fluctuations. Respectively, in neuropsychiatric disorders, alterations in hub connectivity at specific frequency sub-bands of the BOLD signal could reflect impairments in specific integrational processes which might affect specific brain functions. However, as we operate on data acquired during resting-state, we cannot make direct inferences about the relationship between frequency-specific regional connectivity and brain function. Nonetheless, we believe that the differentiated, frequency-specific hub profiles of distinct brain regions have different functional relevance and support distinct brain processes at the given timescales of BOLD activity. It would be highly interesting to further investigate the relationship between frequency-specific spatial distribution of functional hubs and brain function, as well as behavioral traits.

### Frequency-Specific Decreases in Regional Hubness in Major Depressive Disorder

Major depressive disorder has been associated with widespread alterations in FC across multiple large-scale neural systems. Previous research highlighted aberrant connectivity of the DMN, SN, and CEN as prominent features of MDD (Sheline et al., 2009; Menon, 2011; Hamilton et al., 2013; Li et al., 2013; Manoliu et al., 2013; Sambataro et al., 2013). In addition, altered connectivity of the visual, sensorimotor, and auditory networks has been reported (Veer et al., 2010; Zeng et al., 2012b). More recently, frequency-specific alterations in resting-state activity have been examined in MDD patients. Luo et al. (2015) revealed frequency-dependent aberrant nodal centralities in the DMN, CEN and visual network at frequency band 0.03–0.06 Hz. Xue et al. (2016) found changes in regional homogeneity (ReHo) in the middle occipital gyrus, ACC, inferior and superior frontal gyrus, mPFC, and thalamus across frequency bands 0.01–0.027 Hz and 0.027–0.073 Hz. At the same frequency bands, Wang et al. (2016) found altered amplitude of low-frequency fluctuations (ALFF) in the ventromedial prefrontal cortex, inferior frontal gyrus, precentral gyrus, posterior cingulate and precuneus.

In this study, we investigated frequency-resolved alterations in regional degree centrality in MDD patients as compared to healthy controls under the consideration of the broad spectrum of accessible frequencies (i.e., 0.01–0.25 Hz) and at a much more fine-grained spectral resolution.

To control for the possible effects of GM atrophy on the DC measure, we performed a VBM analysis and an additional analysis of group-differences in DC under consideration of GMV reduction in MDD patients. Reduced GMV was observed in the left dorsal ACC, left hippocampus, and bilateral middle frontal gyrus – findings which are consistent with previous reports of GM abnormalities in major depression (Drevets et al., 2008; Koolschijn et al., 2009; Liu et al., 2010, 2017; Kempton et al., 2011; Grieve et al., 2013; Lai, 2013). However, in MDD patients, the GMV reductions did not critically impact changes in regional DC. This observation highlights that although there are systematic changes in regional GMV in MDD, they do not necessarily affect the region’s FC profile. GMV atrophy in MDD – although often implicated in the disease – is not the primary characteristic of it. It is possible that the observed GMV reductions are too subtle to affect the overall regional connectivity or its representation in fMRI. Thus, it seems that the observed regional FC changes in MDD are rather attributable to alterations in the intrinsic dynamics of the BOLD signal, than to changes in GMV. Altogether, the GMV correction helps to elucidate that FC changes occur independently of structural changes, and points toward other mechanisms – related to the underlying neuronal activity – that affect the DC measure.

Importantly – independently of regional GM atrophy – we found widespread frequency-specific alterations of regional hubness in MDD patients. At the lowest frequency band (freq1: 0.01–0.025 Hz) decreased DC of the transverse temporal gyrus and the insula was observed. At the middle-frequency band 4 (0.075–0.1 Hz), the middle occipital gyrus and the calcarine gyrus exhibited decreased overall connectivity. At middle-frequency bands 5–7 (0.1–0.175 Hz) the supramarginal gyrus, precuneus, angular gyrus, and MCC were largely affected. At higher frequency bands 8–10 (0.175–0.0.25 Hz) the ventral and dorsal ACC was affected, as well as the IPL and supramarginal gyrus. Interestingly, distinct parts of the insula exhibited decreased DC at different frequency bands, including low- middle- and high frequencies. Reduced DC of the left angular gyrus at frequency band 5 (0.1–0.125 Hz), as well as of the left superior temporal gyrus and the posterior insula at frequency band 9 (0.2–0.225 Hz), was negatively correlated with depression symptoms such as the BDI score and the length of current episode, respectively. Altogether, brain regions which exhibited a significantly reduced DC in MDD patients could be attributed to the DMN, SN, as well as the visual, sensorimotor, and auditory networks – which is in line with previous findings on brain functional changes in MDD.

The frequency-resolved analysis proved to be a more specific method to detect alterations in regional hubness in MDD, since the analysis of group differences in DC both within the full-band (i.e., 0.01–0.25 Hz) as well as within the conventional band (i.e., 0.01–0.1 Hz) of BOLD signal frequencies did not reveal any significant changes at the given statistical threshold. Thus, it is important to consider the frequency content when analyzing resting-state functional connectivity patterns, and alterations in MDD. Discrepant findings of altered FC in MDD across studies could occur due to different frequency content of fMRI signal under investigation.

Notably, we only observe pathological decreases in regional hubness in MDD, but no increases in regional hubness. By the example of the dynamic core network, central hubness relates to dynamic integration from diverse sources and promotes flexible, time-varying topological states for highly efficient information transfer. Decreased hubness might result in the loss of such flexibility and the reduced variability in entering distinct connectivity states, which in turn might translate to the pathophysiology of MDD. A recent model conceptualizes MDD as arising from imbalanced state shift, in which patients are stuck in a state of negative mood (Holtzheimer and Mayberg, 2011). Resting-state fMRI studies in MDD showed a decreased variability in network FC (Demirtaş et al., 2016), and a prolonged occurrence of certain dynamic FC states (Allen et al., 2014; Calhoun et al., 2014), which can be linked to ruminative behavior (Kaiser et al., 2016; Zhi et al., 2018).

Below, we interpret the regions of decreased DC at the network level and discuss the putative implications of decreased hubness in brain regions of the DMN, SN, as well as the visual, sensorimotor, and auditory networks on the pathophysiology and symptomatology of MDD.

#### Default-Mode Network

The DMN is involved in self-referential and internally oriented processes (Buckner et al., 2008). In the context of the pathophysiology and symptomology of MDD, increased FC within DMN has been linked to pathological ruminative behavior, where patients cannot disengage from internal mental processing of emotionally salient events (Berman et al., 2011; Zhu et al., 2012, 2017). In the current study, we observe a decreased hubness of the DMN at a broad range of middle- to high-frequency bands. The ventral ACC – part of the anterior DMN – exhibited reduced hubness exclusively at high frequencies. A loss of hubness in the ventral ACC has been reported before by Wu et al. (2016). Moreover, our results yielded a significant correlation between the decreased hubness of the posterior DMN subdivision (i.e., the angular gyrus) at middle frequencies and the depressive symptoms. Decreased hubness of the DMN in MDD could reflect increased intra-modular connectivity (i.e., FC within the DMN itself) at the expense of inter-modular connectivity (i.e., FC to other networks).

#### Salience Network

The SN is anchored in the bilateral anterior insula (AI) and the dorsal anterior cingulate cortex (dACC), but also includes three key subcortical structures: the amygdala, the ventral striatum, and the substantia nigra/ventral tegmental area (VTA). The SN plays a key role in saliency detection, and through its extensive subcortical connections, in emotional control (Menon and Uddin, 2010; Uddin, 2014; Menon, 2015).

The insula is believed to be highly relevant to neuropsychiatric disorders which entail deficits in higher order cognitive, emotional and social processing. Studies propose a tripartite model of insula functional subdivisions, dividing it into dorsal-anterior, ventral-anterior, and posterior part (Deen et al., 2011; Kelly et al., 2012; Chang et al., 2013). Altogether, the insula constitutes a key hub for meta-awareness and affective processing (Craig, 2009b; Chang et al., 2013). The right anterior insula is a key node of the SN and initiates network switching between the DMN and CEN, thus, dynamically gates saliency allocation and behavioral response toward either internally or externally driven content (Sridharan et al., 2008; Menon and Uddin, 2010; Goulden et al., 2014). Due to its subjective and self-referential nature, however, the representation and perception of saliency can be vastly disrupted in psychopathology. Indeed, MDD patients exhibit (i) altered insular structure (Liu et al., 2010; Takahashi et al., 2010; Peng et al., 2011); (ii) altered insular resting-state connectivity (Liu et al., 2010; Manoliu et al., 2013; Avery et al., 2014; Iwabuchi et al., 2014; Ambrosi et al., 2017; Peng et al., 2018); (iii) elevated insular reactivity to negatively valenced stimuli (Lee et al., 2008); (iv) decreased insular reactivity to exteroceptive stimuli (leading to a predominance of interoceptive stimulus processing; Wiebking et al., 2010) alongside abnormal interoceptive representation (Avery et al., 2014). These abnormalities can altogether be associated with a bias toward negative thoughts and self-image (Northoff, 2007), and failure in the exertion of cognitive control over emotional processing. The insula also plays a key role in time perception (Craig, 2009a; Wittmann, 2009) and its altered intrinsic activity could relate to distorted time perception in depression patients (Bschor et al., 2004; Mahlberg et al., 2008; Fuchs, 2013; Thönes and Oberfeld, 2015; Northoff, 2016; Stanghellini et al., 2017).

Our finding of decreased DC of the insula in MDD patients relates to the above reports. Moreover, the observed frequency-specific alterations in distinct parts of the insula correspond to findings which show that the insular subdivisions are differentially affected in depression (Peng et al., 2018), and support the notion that distinct processes are carried out at different frequencies of the neural signal (Penttonen and Buzsáki, 2003; Draguhn and Buzsáki, 2004; Knyazev, 2007). Furthermore, our findings corroborate recent reports on frequency-dependent hub role of distinct subdivisions of the right anterior insula (Wang et al., 2018).

Decreased DC of the dorsal ACC – observed exclusively at higher frequencies – could further contribute to impairments in saliency processing and deficits in attentional control over emotional stimuli.

#### Visual Network

In the current study, we observe a decreased overall connectivity of the occipital cortex at frequency band 4 (0.075–0.1 Hz). The visual system has been widely reported to be implicated in depression. Altered excitatory (glutamate) and inhibitory (GABA) neurotransmitter levels in the occipital cortex were observed in MDD (Sanacora et al., 2004) implicating an overactive occipital system, while efficient treatment was shown to bring GABA to pre-symptomatic levels (Sanacora et al., 2002; Sanacora et al., 2003). Correspondingly, in a recent study of ours, we showed that MDD is associated with pathologically increased resting-state BOLD activity within a secondary-occipital visual network (Ries et al., 2018). Further functional alterations within the visual system in MDD have been reported by fMRI studies (Veer et al., 2010; Zeng et al., 2012b) and may be linked to social avoidance (Yao et al., 2009; Liu et al., 2010), as well as impaired selective attention and working memory (Le et al., 2017) in MDD patients.

#### Sensorimotor Network

In the current analysis, we also observe decreased DC of the left precentral gyrus – a region associated with the sensorimotor network – within one of the middle-frequency bands (freq5; 0.1–0.125 Hz). Several reports of altered structure (Taki et al., 2005; Zeng et al., 2012a; Grieve et al., 2013; Peng et al., 2015) and function (Veer et al., 2010; Tsujii et al., 2017) of the precentral gyrus have been made in MDD. In depressive patients with suicidal idealization, altered structure and function of the precentral gyrus has been associated with malfunctioning impulsivity control, i.e., inhibitory control over exerted actions (Tsujii et al., 2017). Psychomotor functions are speculated to be strongly altered in depression: “the decreased environment-focus may also be manifest in lack of motivation and volition to act in the external environment which ultimately may result in psychomotor retardation and social withdrawal on the psychopathological side” (Northoff, 2016).

#### Auditory Network

At the lowest frequency band (freq1; 0.01–0.025 Hz), we observed decreased DC of the right transverse temporal gyrus (or Heschl’s gyrus) within the primary auditory cortex. Decreased resting-state FC of the Heschl’s gyrus has been reported in depression before (Veer et al., 2010).

## Conclusion

In conclusion, results of our current study reveal a frequency-dependent spatial organization of functional hubs in the human brain. MDD is associated with frequency-specific decreases in regional hubness, which is independent of GM atrophy. The overall connectivity (i.e., degree centrality) of brain areas associated with the default-mode network, salience network, as well as sensorimotor, visual, and auditory networks was found to be decreased at specific frequency bands. The frequency-resolved signal analysis proved to be a more sensitive method for detecting disease-related alterations in regional hubness as compared to signal analysis at the full frequency range. Importantly, our results stress the need for considering higher frequencies of the BOLD signal (i.e., >0.1 Hz), as they hold unique information about functional organization of the brain at rest. Altogether, our findings highlight the importance of frequency content when examining brain organizational properties in health, and its alterations in major depressive disorder.

## Ethics Statement

This study was carried out in accordance with the Human Research Committee guidelines of the Klinikum rechts der Isar, Technische Universität München with written informed consent from all subjects in accordance with the Declaration of Helsinki.

## Author Contributions

AR and AW conceived the research. AR, CM, and MH preprocessed the data. AR analyzed the data, prepared the figures, and drafted the manuscript. AR, MH, CS, and AW edited and revised the manuscript. AR, MH, SG, CM, CS, and AW approved final version of manuscript.

## Conflict of Interest Statement

The authors declare that the research was conducted in the absence of any commercial or financial relationships that could be construed as a potential conflict of interest.

## Footnotes

1 http://www.fil.ion.ucl.ac.uk/spm/

2 https://www.nitrc.org/projects/pestica/

3 http://restfmri.net/forum/REST

4 http://www.neuro.uni-jena.de/cat/
