# Supplementary material for: Frequency-Dependent Spatial Distribution of Functional Hubs in the Human Brain and Alterations in Major Depressive Disorder
Source: Front Hum Neurosci. 2019 May 14;13:146. doi: 10.3389/fnhum.2019.00146 (PMC6527901; doi:10.3389/fnhum.2019.00146)
Supplement: Supplementary file 1 [file Data_Sheet_1.PDF]

Supplementary Material

Frequency-dependent spatial distribution of functional hubs in the human brain and alterations in major depressive disorder.

Anja Ries[1,2], Matthew Hollander[1,2], Sarah Glim[1,2,3], Chun Meng[1,2,4], Christian Sorg[1,2,5], Afra Wohlschläger[1,2 *]

[1]Department of Neuroradiology, Technical University of Munich TUM, Munich, Germany

[2]TUM-Neuroimaging Center, Technical University of Munich TUM, Munich, Germany

[3]Graduate School of Systemic Neurosciences, LMU Munich, Planegg-Martinsried, Germany

[4]Department of Psychiatry, University of Cambridge, Cambridge, United Kingdom

[5]Department of Psychiatry, Technical University of Munich TUM, Munich, Germany

Table of Contents

1. ***Figures***
   - *Figure S1. Group differences in gray matter volume* 2
   - *Figure S2. Group differences in GMV-corrected DC at 10 frequency bands* 9
2. ***Tables***
   - *Table S1. Group differences in gray matter volume* 2
   - *Table S2a. DC in healthy controls at 10 frequency bands* 3
   - *Table S2b. DC in healthy controls at full frequency range* 6
   - *Table S2c. DC in healthy controls at conventional frequency range* 7
   - *Table S3. Group differences in DC at 10 frequency bands* 7
   - *Table S4. Group differences in GMV-corrected DC at 10 frequency bands* 10

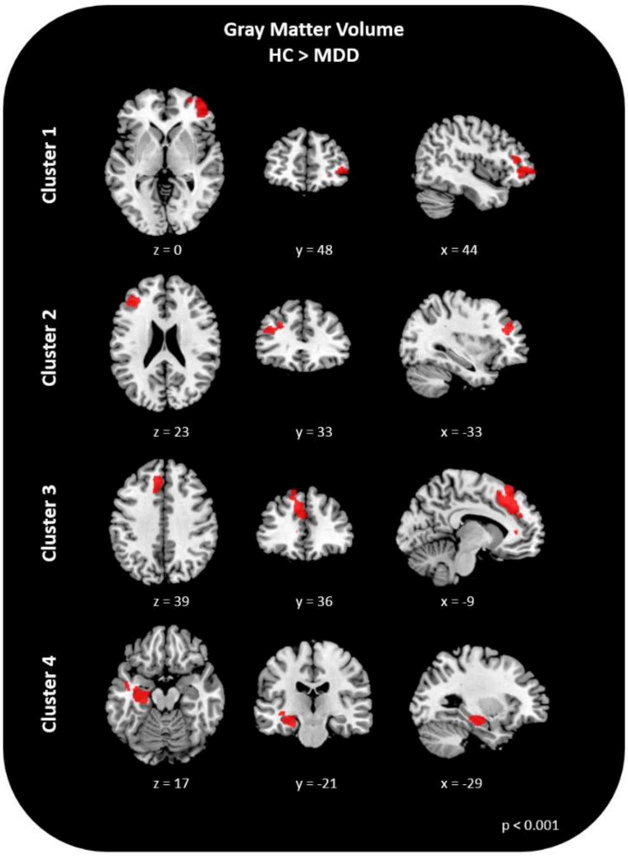

Figure S1. *Regional differences in gray matter volume (GMV) between groups (p cluster-level corrected < 0.05, on an underlying voxel-level correction of p < 0.001). The analysis yielded four clusters of decreased GMV in MDD patients as compared to healthy controls (HC). Cluster 1: right middle frontal gyrus & right middle orbital gyrus; cluster 2: left middle frontal gyrus; cluster 3: left superior medial frontal gyrus & left anterior cingulate cortex (ACC); cluster 4: left hippocampus & left superior temporal gyrus.*

Table S1. Results of the VBM group comparison analysis, including the cluster size and the z-value, MNI coordinates, side, and anatomical locations of the peak voxels. Reported are only brain areas with p cluster-level corrected < 0.05, on underlying voxel-level correction of p < 0.001. Significant group differences in gray matter volume (GMV) were found only in the HC > MDD contrast.

GMV: HC > MDD		MNI coordinates [mm]				
cluster size	z-value	x	y	z	side	location
1032	4.44	44	48	0	R	Middle Frontal Gyrus
	3.85	30	54	-3	R	Middle Orbital Gyrus
872	4.11	-39	41	29	L	Middle Frontal Gyrus
2000	4.37	-8	32	32	L	Superior Medial Frontal Gyrus
	4.29	-3	36	29	L	ACC
1473	4.07	-29	-21	-17	L	Hippocampus
	4.05	-47	-5	-14	L	Superior Temporal Gyrus

Table S2a. Results of the degree centrality (DC) analysis in healthy controls (HC) at 10 frequency bands, including the cluster size and the z-value, MNI coordinates, side, and anatomical locations of the peak voxels. Reported are only brain areas with p cluster-level corrected < 0.05, on underlying voxel-level correction of p < 0.001, with voxels restricted to gray matter.

DC: HC			MNI coordinates [mm]				
frequency band	cluster size	z-value	x	y	z	side	location
freq1	240	5.14	-39	-40	56	L	Postcentral Gyrus
	400	5.91	15	-85	38	R	Cuneus
		5.46	21	-79	38	R	Superior Occipital Gyrus
	118	5.57	12	-28	65	R	Paracentral Lobule
		4.51	24	-49	65	R	Superior Parietal Lobule
	21	5.36	51	-46	20	R	Superior Temporal Gyrus
	190	5.11	-18	-16	71	L	Precentral Gyrus
		4.52	-3	-25	50	L	Paracentral Lobule
	121	5.11	-63	-13	5	L	Superior Temporal Gyrus
		4.63	-60	-25	26	L	SupraMarginal Gyrus
	87	4.96	-45	-70	2	L	Middle Occipital Gyrus
		4.13	-42	-64	20	L	Middle Temporal Gyrus
	69	4.94	48	-61	8	R	Middle Temporal Gyrus
	62	4.86	42	-13	53	R	Precentral Gyrus
		4.38	54	-22	50	R	Postcentral Gyrus
	45	4.8	-24	-79	26	L	Superior Occipital Gyrus
		4.29	-33	-88	23	L	Middle Occipital Gyrus
	60	4.69	54	-19	17	R	Rolandic Operculum
		4.68	54	-16	8	R	Superior Temporal Gyrus
	24	4.59	42	-31	14	R	Superior Temporal Gyrus
	40	4.32	48	8	-1	R	Insular Lobe
		4.3	60	2	5	R	Superior Temporal Gyrus
	38	4.11	21	-82	-10	R	Lingual Gyrus
		3.71	6	-94	-4	R	Calcarine Gyrus
freq2	378	5.99	12	-73	26	R	Cuneus
		4.83	-3	-64	8	L	Calcarine Gyrus
	68	5.27	-45	-4	53	L	Precentral Gyrus
		3.66	-60	-19	32	L	Postcentral Gyrus
	85	4.95	-18	-19	71	L	Precentral Gyrus
		4.85	-24	-7	65	L	Superior Frontal Gyrus
	349	5.21	-12	-37	44	L	MCC
		4.91	3	8	44	R	MCC
		4.89	-3	-25	50	L	Paracentral Lobule
	45	5.12	42	-13	53	R	Precentral Gyrus
		4.18	45	-1	53	R	Middle Frontal Gyrus
	38	4.97	48	-58	8	R	Middle Temporal Gyrus
	102	4.95	24	-40	62	R	Postcentral Gyrus
		3.87	24	-28	71	R	Precentral Gyrus
	121	4.51	57	-22	17	R	Superior Temporal Gyrus
		4.5	57	8	2	R	Rolandic Operculum
		4.45	54	-7	5	R	Transverse Temporal Gyrus
	24	4.34	-57	-4	2	L	Superior Temporal Gyrus
	40	4.31	-51	-67	5	L	Middle Temporal Gyrus
		4.14	-45	-76	2	L	Middle Occipital Gyrus
	33	3.98	-24	-46	65	L	Superior Parietal Lobule
	25	3.55	24	-1	65	R	Superior Frontal Gyrus
freq3	146	5.79	60	-16	11	R	Superior Temporal Gyrus
		5.23	51	-19	8	R	Transverse Temporal Gyrus

		4.47	63	-31	32	R	SupraMarginal Gyrus
	60	5.71	-48	-4	47	L	Precentral Gyrus
		4.96	-51	-13	47	L	Postcentral Gyrus
		3.85	-39	2	50	L	Middle Frontal Gyrus
	325	5.08	3	-82	17	L	Cuneus
	58	5.08	42	2	56	R	Middle Frontal Gyrus
	170	4.95	3	-40	47	R	MCC
		4.82	-3	-28	50	L	Paracentral Lobule
	31	4.91	-45	-46	53	L	Inferior Parietal Lobule
		3.8	-39	-46	59	L	Superior Parietal Lobule
	31	4.89	-57	-13	11	L	Superior Temporal Gyrus
		4.66	-54	-1	5	L	Rolandic Operculum
	59	4.86	30	-49	62	R	Superior Temporal Lobule
		4.12	24	-34	68	R	Postcentral Gyrus
	35	4.59	54	-46	14	R	Superior Temporal Gyrus
	26	4.51	-42	-19	-1	L	Superior Temporal Gyrus
	27	4.34	-57	-34	17	L	Superior Temporal Gyrus
	24	4.11	54	5	-1	R	Temporal Pole
		3.65	42	11	5	R	IFG (p. Opercularis)
	29	4.14	-15	8	65	L	Superior Frontal Gyrus
	27	4.04	51	-55	8	R	Middle Temporal Gyrus
freq4	1214	6.78	0	-76	20	L	Cuneus
		5.76	6	-85	23	R	Cuneus
	101	6.21	27	-82	26	R	Superior Occipital Gyrus
	411	5.95	-3	-43	50	L	MCC
		5.94	0	-49	35	L	Precuneus
	82	5.77	-15	11	65	L	Superior Frontal Gyrus
		3.86	-27	-4	56	L	Precentral Gyrus
	171	5.65	39	-16	-4	R	Insular Lobe
		5.48	51	-16	5	R	Transverse Temporal Gyrus
		4.86	57	5	2	R	Rolandic Operculum
	21	5.56	-57	2	2	L	Rolandic Operculum
	284	5.52	57	-49	23	R	Superior Temporal Gyrus
		5.24	51	-73	17	R	Middle Temporal Gyrus
	280	5.48	-51	-43	50	L	Inferior Parietal Lobule
		5.46	-45	-1	50	L	Precentral Gyrus
		5.3	-51	-16	47	L	Postcentral Gyrus
	62	5.36	-63	-13	5	L	Superior Temporal Gyrus
		3.78	-54	-16	14	L	Postcentral Gyrus
	23	4.83	6	41	14	R	ACC
	30	4.51	42	-25	17	R	Rolandic Operculum
	20	3.23	57	-1	41	R	Precentral Gyrus
freq5	1953	5.96	-42	-76	26	L	Middle Occipital Gyrus
		5.65	0	-82	32	L	Cuneus
		5.62	12	-79	38	R	Cuneus
	103	4.68	-45	5	38	L	Precentral Gyrus
		4.3	-48	8	29	L	IFG (p. Opercularis)
	91	4.53	-51	-10	5	L	Superior Temporal Gyrus
	34	4.5	48	-73	17	R	Middle Temporal Gyrus
	55	4.42	48	11	-1	R	Insular Lobe
		4	57	5	2	R	Rolandic Operculum
		3.77	63	-4	2	R	Superior Temporal Gyrus
	78	4.34	63	-31	32	R	SupraMarginal Gyrus
		3.97	54	-49	26	R	Angular Gyrus
	59	4.18	36	-43	53	R	Inferior Parietal Lobule
		4.08	33	-46	44	R	Postcentral Gyrus

freq6	1194	5.89	3	-43	44	R	Precuneus
		5.74	-6	-82	35	L	Cuneus
		5.63	6	-52	35	R	MCC
	531	5.28	-48	-43	53	L	Inferior Parietal Lobule
		5.25	-45	-58	20	L	Middle Temporal Gyrus
	29	4.75	-45	-7	50	L	Precentral Gyrus
	112	4.68	57	-46	32	R	SupraMarginal Gyrus
		4.25	54	-58	32	R	Angular Gyrus
	54	4.63	-27	-82	23	L	Middle Occipital Gyrus
	26	4.3	33	-88	11	R	Middle Occipital Gyrus
	38	4.08	51	-10	2	R	Superior Temporal Gyrus
		3.39	51	5	-7	R	Temporal Pole
freq7	963	6.06	3	-40	47	R	MCC
		5.77	-6	-76	29	L	Cuneus
		5.49	0	-58	44	L	Precuneus
	415	5.88	-51	-61	32	L	Angular Gyrus
		5.76	-45	-67	23	L	Middle Temporal Gyrus
		5.71	-45	-64	44	L	Angular Gyrus
	228	4.9	36	-67	44	R	Angular Gyrus
		4.5	57	-49	26	R	SupraMarginal Gyrus
		4.24	45	-52	44	R	Inferior Parietal Lobule
	21	4.76	51	-16	8	R	Transverse Temporal Gyrus
	54	4.68	-57	-1	2	L	Rolandic Operculum
		4	-48	-16	11	L	Transverse Temporal Gyrus
	35	4.25	42	2	53	R	Middle Frontal Gyrus
		4.05	45	-7	56	R	Precentral Gyrus
freq8	91	5.31	54	-19	8	R	Superior Temporal Gyrus
	169	4.81	-3	-70	47	L	Precuneus
		4.27	0	-73	26	L	Cuneus
	170	4.77	-45	-67	38	L	Angular Gyrus
		4.57	-42	-73	26	L	Middle Occipital Gyrus
	81	4.54	-3	38	14	L	ACC
		3.94	6	47	14	R	ACC
	75	4.43	-51	-10	5	L	Superior Temporal Gyrus
		4.03	-54	8	11	L	IFG (p. Opercularis)
freq9	591	6.35	-12	-70	26	L	Cuneus
		5.77	3	-40	47	R	MCC
		5.62	-3	-64	47	L	Precuneus
	517	5.32	-54	-52	32	L	Angular Gyrus
		5.24	-42	-40	50	L	Inferior Parietal Lobule
		5.22	-45	-64	23	L	Middle Temporal Gyrus
	110	5.1	57	2	-1	R	Superior Temporal Gyrus
		4.49	42	-13	-1	R	Insular Lobe
	76	4.85	-51	-10	8	L	Transverse Temporal Gyrus
		4.13	-42	-16	-4	L	Superior Temporal Gyrus
		3.59	-54	5	2	L	Rolandic Operculum
	49	4.35	9	47	5	R	ACC
		3.98	0	41	14	L	ACC
freq10	400	5.9	0	-40	44	L	MCC
		5.36	0	-67	47	L	Precuneus
	253	5.11	-45	-43	50	L	Inferior Parietal Lobule
		5	-42	-76	26	L	Middle Occipital Gyrus
		4.89	-45	-70	35	L	Angular Gyrus
	41	4.56	-54	-13	5	L	Superior Temporal Gyrus
		3.37	-54	-1	5	L	Rolandic Operculum
	20	4.11	60	-49	29	R	SupraMarginal Gyrus

	3.53	57	-52	38	R	Inferior Parietal Lobule
53	3.89	-3	35	17	L	ACC

Table S2b. Results of the degree centrality (DC) analysis in healthy controls (HC) at the full frequency range (0.01–0.25 Hz), including the cluster size and the z-value, MNI coordinates, side, and anatomical locations of the peak voxels. Reported are only brain areas with p cluster-level corrected < 0.05, on underlying voxel-level correction of p < 0.001, with voxels restricted to gray matter.

DC: HC			MNI coordinates [mm]				
	cluster size	z-value	x	y	z	side	location
full frequency range 0.01-0.25 Hz	53	5.53	-45	-76	20	L	Middle Temporal Gyrus
		4.25	-36	-88	8	L	Middle Occipital Gyrus
	41	5.49	-45	-4	50	L	Precentral Gyrus
	34	5.38	-42	-43	56	L	Inferior Parietal Lobule
	126	5.14	-3	-25	50	L	Paracentral Lobule
		4.73	9	-31	44	R	MCC
	322	5.01	3	-79	17	L	Cuneus
		4.77	30	-79	29	R	Middle Occipital Gyrus
		4.72	12	-76	35	R	Cuneus
	45	4.73	57	-19	17	R	Rolandic Operculum
		4.43	51	-16	8	R	Transverse Temporal Gyrus
		3.54	66	-16	8	R	Superior Temporal gyrus
	46	4.71	48	11	-1	R	Insular Lobe
		4.64	60	5	-1	R	Temporal Pole
		3.66	60	5	11	R	Rolandic Operculum
	36	4.69	-60	-13	5	L	Superior Temporal Gyrus
		3.7	-48	-16	14	L	Rolandic Operculum
	35	4.43	51	-13	50	R	Precentral Gyrus
		3.71	54	-22	50	R	Postcentral Gyrus
		3.42	45	-4	56	R	Middle Frontal Gyrus
	33	4.3	-54	-34	20	L	Superior Temporal Gyrus
		3.8	-54	-46	26	L	SupraMarginal Gyrus
	34	4.17	54	-55	26	R	Angular Gyrus
		3.68	57	-49	20	R	Superior Temporal Gyrus
		3.3	57	-43	29	R	SupraMarginal Gyrus
	21	4.11	60	-37	32	R	SupraMarginal Gyrus
	21	3.96	51	-61	8	R	Middle Temporal Gyrus
	42	3.95	3	-55	41	R	Precuneus
		3.83	0	-67	47	L	Precuneus
	20	3.81	-54	-52	32	L	Angular Gyrus
	18	3.78	-27	-46	65	L	Superior Parietal Lobule
	19	3.72	-30	-82	26	L	Middle Occipital Gyrus

Table S2c. Results of the degree centrality (DC) analysis in healthy controls (HC) at the conventional frequency range (0.01–0.1 Hz), including the cluster size and the z-value, MNI coordinates, side, and anatomical locations of the peak voxels. Reported are only brain areas with p cluster-level corrected < 0.05, on underlying voxel-level correction of p < 0.001, with voxels restricted to gray matter.

DC HC			MNI coordinates [mm]				
	cluster size	Z value	x	y	z	Side	Location
conventional frequency range 0.01-0.1 Hz	83	5.99	-48	-10	50	L	Postcentral Gyrus
		3.73	-45	2	38	L	Precentral Gyrus
	321	5.87	-3	-28	50	L	Paracentral Lobule
		4.55	9	-31	44	R	MCC
	408	5.55	3	-79	17	L	Cuneus
		4.82	24	-82	35	R	Superior Occipital Gyrus
		4.79	15	-73	26	R	Cuneus
	35	5.3	-63	-13	5	L	Superior Temporal Gyrus
		3.99	-54	-1	2	L	Rolandic Operculum
		3.73	-60	-13	-4	L	Middle Temporal Gyrus
	25	5.16	-42	-43	56	L	Inferior Parietal Lobule
	54	5.15	-45	-76	20	L	Middle Temporal Gyrus
		4.32	-48	-73	5	L	Middle Occipital Gyrus
	44	4.89	48	-58	8	R	Middle Temporal Gyrus
	92	4.89	-18	-19	71	L	Precentral Gyrus
		4.51	-21	-4	68	L	Superior Frontal Gyrus
	22	4.89	-30	-37	65	L	Postcentral Gyrus
	104	4.84	27	-49	65	R	Superior Parietal Lobule
		4.53	27	-43	59	R	Postcentral Gyrus
	198	4.79	57	-19	17	R	Rolandic Operculum
		4.73	48	11	-1	R	Insular Lobe
		4.5	39	-28	17	R	Transverse Temporal Gyrus
	21	4.61	39	-16	-1	R	Insular Lobe
	57	4.45	51	-13	50	R	Precentral Gyrus
	41	4.45	51	-46	20	R	Superior Temporal Gyrus
		4.12	57	-43	29	R	SupraMarginal Gyrus
	18	4.3	12	-64	65	R	Precuneus
	24	3.77	-30	-43	65	L	Postcentral Gyrus
	30	4.03	60	-31	35	R	SupraMarginal Gyrus
	45	3.96	6	2	71	R	Superior Frontal Gyrus
		3.94	15	2	68	R	Medial Frontal Gyrus
		3.46	30	-4	53	R	Middle Frontal Gyrus
	30	3.67	-60	-34	26	L	SupraMarginal Gyrus
		3.62	-51	-34	20	L	Superior Temporal Gyrus

Table S3. Results of the degree centrality (DC) group comparison analysis at 10 frequency bands including the cluster size and the z-value, MNI coordinates, side, and anatomical locations of the peak voxels. Reported are only brain areas with p cluster-level corrected < 0.05, on underlying voxel-level correction of p < 0.001, with voxels restricted to gray matter. Significant group differences in DC were found only in the HC > MDD contrast.

DC: HC > MDD			MNI coordinates [mm]				
frequency band	cluster size	z-value	x	y	z	side	location
freq1	23	4.74	42	-19	5	R	Transverse Temporal Gyrus
	38	4.05	45	5	-4	R	Insular Lobe

		3.6	45	8	8	R	IFG (p. Opercularis)
freq2	50	5.14	42	5	8	R	Insular Lobe
freq4	31	4.87	-36	-85	5	L	Middle Occipital Gyrus
	26	4.78	3	-76	17	L	Calcarine Gyrus
	27	4.31	-45	-16	-4	L	Superior Temporal Gyrus
	30	3.97	51	2	2	R	Rolandic Operculum
		3.88	42	5	-7	R	Insular Lobe
freq5	57	4.96	3	-10	35	R	MCC
		3.81	-6	-16	38	L	MCC
	43	4.65	42	2	-7	R	Insular Lobe
		4.35	42	8	8	R	IFG (p. Opercularis)
		4.15	54	-4	2	R	Superior Temporal Gyrus
	44	4.52	-51	-52	32	L	Angular Gyrus
		3.42	-57	-43	26	L	SupraMarginal Gyrus
		3.29	-57	-52	17	L	Middle Temporal Gyrus
	32	4.31	-45	8	38	L	Precentral Gyrus
	24	4.21	63	-46	29	R	SupraMarginal Gyrus
		3.79	57	-52	26	R	Angular Gyrus
freq6	30	5.12	60	-43	26	R	SupraMarginal Gyrus
	30	4.52	0	-13	38	L	MCC
	22	3.89	6	-46	59	R	Precuneus
freq7	34	5.22	-9	-43	35	L	MCC
		4.21	-12	-43	44	L	Precuneus
	31	4.47	-42	-22	-1	L	Insular Lobe
		3.64	-48	-13	-4	L	Superior Temporal Gyrus
	89	4.58	-45	-61	44	L	Angular Gyrus
		4.53	-45	-52	53	L	Inferior Parietal Lobule
freq8	57	5.56	36	-13	-7	R	Putamen
		4.52	42	-10	-1	R	Insular Lobe
	157	5.4	-3	35	11	L	ACC
		4.7	9	47	2	R	ACC
	40	4.29	-48	-16	5	L	Superior Temporal Gyrus
	23	4.96	-24	-25	-13	L	Hippocampus
	26	4.3	-57	2	-4	L	Superior Temporal Gyrus
		3.86	-45	14	-16	L	Temporal Pole
	32	4.23	-36	11	-1	L	Insular Lobe
		3.64	-48	20	-4	L	IFG (p. Opercularis)
freq9	43	5.82	6	44	5	R	ACC
		3.49	-3	41	-1	L	ACC
	31	5.1	-51	-13	5	L	Superior Temporal Gyrus
		3.49	-57	-1	2	L	Rolandic Operculum
	25	4.87	54	2	-1	R	Superior Temporal Gyrus
		3.81	57	-1	8	R	Rolandic Operculum
	54	4.81	-54	-49	29	L	SupraMarginal Gyrus
		3.79	-51	-61	23	L	Middle Temporal Gyrus
		3.39	-57	-52	38	L	Inferior Parietal Lobule
	25	4.38	-42	-10	-13	L	Superior Temporal Gyrus
		4.12	-42	-13	-4	L	Insular Lobe
freq10	57	5.25	-6	35	17	L	ACC
		4.32	9	35	20	R	ACC
	27	4.96	-54	-16	8	L	Transverse Temporal Gyrus
		3.87	-54	-28	11	L	Superior Temporal Gyrus

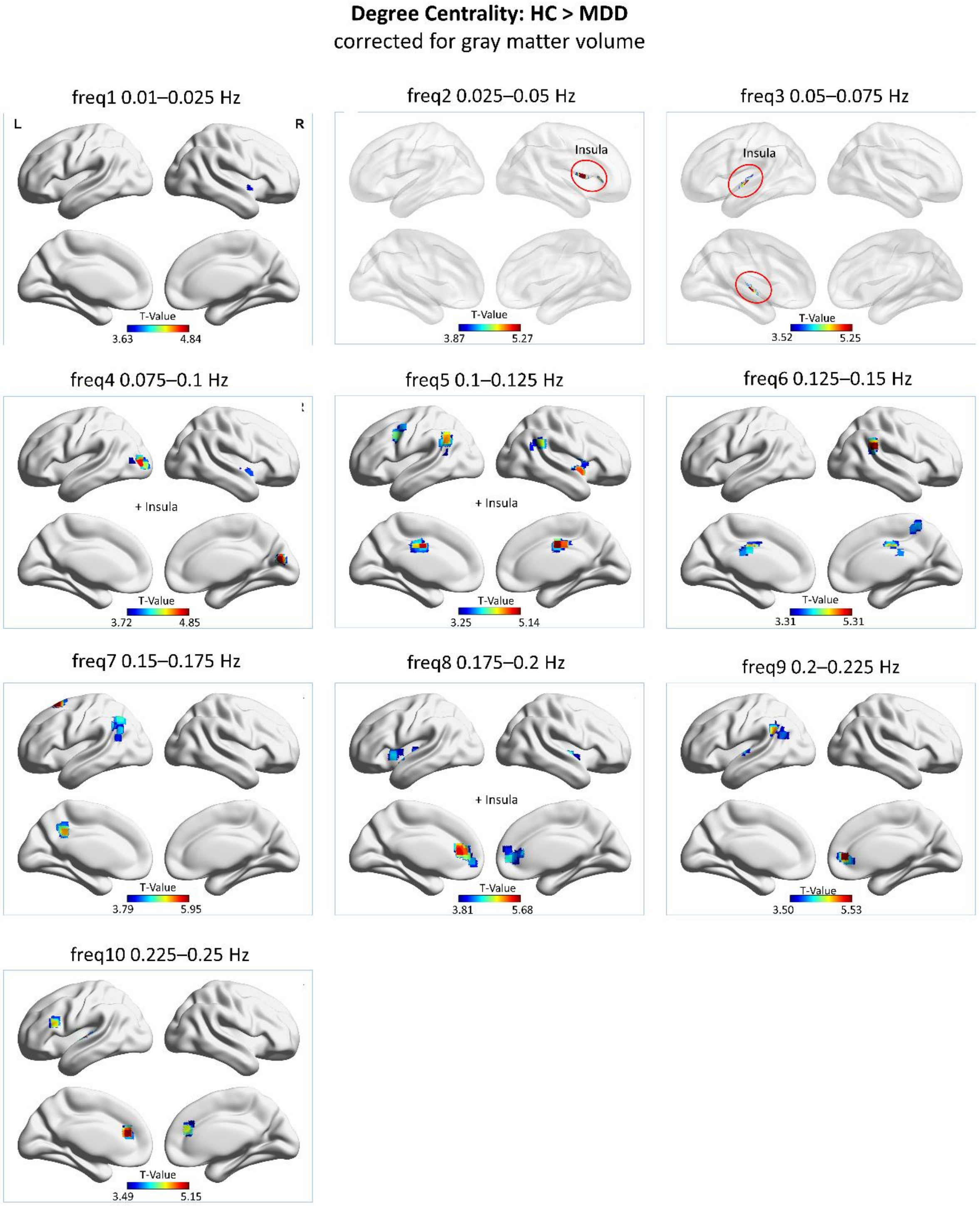

Figure S2. *Group differences in voxel-wise DC across 10 frequency bands controlled for gray matter volume (p cluster-level corrected < 0.05, on underlying voxel-level correction of p < 0.001, restricted with gray matter mask). At freq2 and freq3, the clusters are located exclusively in the Insular Lobe.*

Table S4. Results of the GMV-corrected degree centrality (DC) group comparison analysis at 10 frequency including the cluster size and the z-value, MNI coordinates, side, and anatomical locations of the peak voxels. Reported are only brain areas with p cluster-level corrected < 0.05, on underlying voxel-level correction of p < 0.001, with voxels restricted to gray matter. Significant group differences in DC were found only in the HC > MDD contrast.

DC: HC > MDD, corrected for GMV			MNI coordinates [mm]				
frequency band	cluster size	z-value	x	y	z	side	location
freq1	21	4.77	-45	-16	-1	L	Superior Temporal Gyrus
	37	4.13	45	5	-4	R	Insular Lobe
		3.6	45	8	8	R	IFG (p.Opercularis)
freq2	54	5.18	42	5	8	R	Insular Lobe
freq3	25	5.16	-42	-19	-1	L	Superior Temporal Gyrus
		3.89	-45	-19	8	L	Transverse Temporal Gyrus
freq4	27	4.78	3	-76	17	L	Calcarine Gyrus
	35	4.63	-36	-85	5	L	Middle Occipital Gyrus
	32	3.93	45	2	-1	R	Insular Lobe
		3.82	54	-4	2	R	Superior Temporal Gyrus
freq5	59	5.06	3	-10	35	R	MCC
	38	4.71	42	2	-7	R	Insular Lobe
		4.36	42	8	8	R	IFG (p.Opercularis)
	39	4.57	-51	-52	32	L	Angular Gyrus
		3.35	-57	-52	17	L	Middle Temporal Gyrus
	24	4.32	63	-46	29	R	SupraMarginal Gyrus
		3.72	57	-52	26	R	Angular Gyrus
	31	4.14	-45	5	38	L	Precentral Gyrus
freq6	33	5.22	60	-43	26	R	SupraMarginal Gyrus
	42	4.66	0	-13	38	L	MCC
	21	3.84	6	-46	59	R	Precuneus
		3.77	12	-40	56	R	Paracentral Lobule
freq7	31	5.82	-18	17	62	L	Superior Frontal gyrus
		3.76	-9	11	68	L	Posterior-Medial Frontal
	37	5.22	-9	-43	35	L	MCC
	83	4.64	-45	-61	44	L	Angular Gyrus
		4.45	-45	-55	53	L	Inferior Parietal Lobule
	35	3.78	-45	-16	5	L	Transverse Temporal Gyrus
freq8	50	5.57	36	-13	-7	R	Putamen
		4.48	42	-10	-1	R	Insular Lobe
	137	5.4	-3	35	11	L	ACC
		4.51	9	50	2	R	Superior Medial Gyrus
	57	4.56	-48	-16	5	L	Superior Temporal Gyrus
	30	4.39	-36	11	-4	L	Insular Lobe
		3.82	-42	23	-1	L	IFG (p.Triangularis)
	23	4.32	-54	5	2	L	Rolandic Operculum
		3.87	-45	14	-16	L	Temporal Pole
freq9	30	5.43	6	44	5	R	ACC
	24	5.18	-51	-13	5	L	Superior Temporal Gyrus
	46	4.9	-54	-49	29	L	SupraMarginal Gyrus
		3.93	-51	-61	23	L	Middle Temporal Gyrus
		3.48	-57	-55	38	L	Inferior Parietal Lobule

	21	4.39	-45	-13	-4	L	Superior Temporal Gyrus
freq10	42	5.07	-6	35	17	L	ACC
		4.4	6	38	23	R	ACC
	31	4.93	-54	-16	8	L	Transverse Temporal Gyrus
		3.96	-54	-28	11	L	Superior Temporal Gyrus
